# Supplementary material for: Chromothripsis during telomere crisis is independent of NHEJ, and consistent with a replicative origin
Source: Genome Res. 2019 May;29(5):737–49. doi: 10.1101/gr.240705.118 (PMC6499312; doi:10.1101/gr.240705.118)
Supplement: Supplemental Material [file supp_gr.240705.118_Supplemental_file_1.zip › contigs/annotated_contigs/DB53/contig.3.DB53_length_530_mean_cov_14.9924528302.docx]

**DB53_length_530_mean_cov_14.9924528302**

```
CCCATTTGACAATTTTTGCTGTTGTCGCTATTGCTTTTGGCATCTTGGTCATGAAATCGTCACCCATTCCTATGGCCAGGATGGTATCA
 >chr1:243114973-243115351 - E=2e-206 p=1e-03
CCTAGGTTGTCTTCGAGGGTTTTTGTAGTCTTGGATTTTACATTTAAGTCTTTAATCCATCTTGAGTTGATTTTTGTATATAGTATAAG

GAAGGGGTCAAGTTTCAATCTTCTCCATATGGCTAGCCAGTTATCCCAGCACCATTTACTGAATAGGAAATCCTTTCCCCATTGCTTGT

TCTTGTCAATTTTGTCAAAGATCAGATAGTTAAAGATGTGCAGCTAGCTTGCTTGCCTGCCTGCCTGCCTGCCTTCCTTCCTTCCTTCC

TTTCCTTTCCTTCCTTTCTTTTTCCTT|TCTTTTCCTT|TCTCTTTCTCTCTCTCTCTCTCTCTCTCTCTCTGTCTCTTTC|CTTTCTT
                                      >chr19:13133878-13133920 + E=1e-13 p=0e+00  >chr
TCTTTCTTTCTTTCCTTCCTTCCTTCCTTCCTTCCTTCCTTCCTTCCTTCCTTCCTTCCTTCCTTCGTTCCTTCTTTCCTTCTTTCCT18:626928-627023 - E=2e-39
```
